# Association of dietary fatty acid intake with hypertension in children and adolescents: evidence from the NHANES 2005–2018

**DOI:** 10.3389/fped.2023.1185982

**Published:** 2023-09-11

**Authors:** Xiumin Li, Fengqin Qi, Zhihong Zhao, Jinbang Ma

**Affiliations:** ^1^ Department of Pediatric Medicine, The Second People’s Hospital of Liaocheng, Linqing, China; ^2^ Department of Neurosurgery, The Second People’s Hospital of Liaocheng, Linqing, China

**Keywords:** dietary fatty acids, polyunsaturated fatty acids, hypertension, children and adolescents, NHANES

## Abstract

**Aim:**

This study aims to evaluate the association between dietary fatty acid intake and hypertension in children and adolescents.

**Methods:**

This cross-sectional study used data of children and adolescents aged 8–17 years from the National Health and Nutrition Examination Survey (NHANES) 2005–2018. Dietary intake of total fat and fatty acid was evaluated via two 24-h dietary recall interviews. Multivariate logistic regression models were used to assess the association between fatty acid intake and hypertension, with odds ratios (ORs) and 95% confidence intervals (CIs) calculated. A subgroup analysis was conducted according to gender, age, and body mass index Z-score.

**Results:**

This study included 13,330 subjects, of which 11,614 were non-hypertensive and 1,716 were hypertensive. Higher intake of total polyunsaturated fatty acids (PUFAs) was associated with significantly lower odds of hypertension (OR = 0.85, 95% CI: 0.74–0.97, *P* = 0.018). No significant associations were found between the density of total saturated fatty acid, monounsaturated fatty acids, and PUFAs and the odds of hypertension (all *P* > 0.05). Increased intake of omega-3 (OR = 0.82, 95% CI: 0.72–0.93, *P* = 0.002) and omega-6 (OR = 0.86, 95% CI: 0.75–0.98, *P* = 0.025) PUFAs, octadecatrienoic acid (OR = 0.82, 95% CI: 0.72–0.93, *P* = 0.003), and octadecadienoic acid (OR = 0.86, 95% CI: 0.75–0.98, *P* = 0.025) was associated with significantly lower odds of hypertension, and individuals with higher omega-6/omega-3 ratio had significantly higher odds of hypertension (OR = 1.09, 95% CI: 1.02–1.17, *P* = 0.025). The density of omega-3 PUFAs (OR = 0.86, 95% CI: 0.78–0.95, *P* = 0.004) and octadecatrienoic acid (OR = 0.87, 95% CI: 0.78–0.96, *P* = 0.006) was inversely associated with the odds of hypertension, and the omega-6/omega-3 ratio was positively associated with the odds of hypertension (OR = 1.09, 95% CI: 1.02–1.17, *P* = 0.012).

**Conclusion:**

Total PUFA intake was negatively associated with the odds of hypertension in children and adolescents. Higher intake of omega-3 and omega-6 PUFAs, octadecatrienoic acid, and octadecadienoic acid, as well as density of omega-3 PUFAs and octadecatrienoic acid, was associated with lower odds of hypertension.

## Introduction

An increasing prevalence of hypertension in children and adolescents has become a significant public health issue that drives a considerable amount of research (1). Age and gender differences in this prevalence are considered (2, 3). Intermediate markers of target organ damage, such as left ventricular hypertrophy, carotid artery wall thickening, and retinal vascular changes, can be detected in hypertensive children and adolescents (4). Childhood hypertension is correlated with an increased risk of essential hypertension in adulthood, which then raises the risk of cardiovascular morbidity and mortality (5). The etiology of hypertension in children and adolescents is varied, and this includes a family history of hypertension, an upward trend in obesity, and poor lifestyle choices (6–8). Investigation of the modifiable factors associated with hypertension to facilitate early prevention and control of hypertension among children and adolescents is necessary.

A healthy diet plays an important part in daily life. Intake of fatty acid, such as saturated fatty acids (SFAs), monounsaturated fatty acids (MUFAs), and polyunsaturated fatty acids (PUFAs), which is a modifiable factor, has been reported to be associated with hypertension. Nakamura et al. (9) validated that SFA intake was inversely related to hypertension in the elderly. The association of dietary omega-3 and omega-6 fatty acid intake with the risk of hypertension was confirmed in American adults (10). For adolescents, O’Sullivan et al. (11) evaluated the association between blood pressure (BP) and dietary intake of PUFAs and suggested that gender may influence the relationship between fatty acid intake and BP. A case–control study illustrated that circulating palmitoleic acid was negatively associated with the risk of primary hypertension, and supplementation of exogenous palmitoleic acid could reduce systolic blood pressure (SBP) and improve aortic remodeling by inhibiting NF-*κ*B-mediated inflammation for children and adolescents (12). Reducing SFA intake of children was revealed to lower diastolic blood pressure (DBP) (13). According to the study of Skilton et al., higher intake of omega-3 fatty acid was correlated with decreased BP and prevalence of hypertension in children with low birth weight (14). Limited evidence has been found on the relationship between SFAs, MUFAs, and PUFAs and hypertension among children and adolescents.

This cross-sectional study aimed to assess the association between dietary fatty acid (SFA, MUFA, and PUFA) intake and hypertension in children and adolescents, based on the National Health and Nutrition Examination Survey (NHANES) database. In the present study, PUFAs were divided into two categories: omega-3 PUFA and omega-6 PUFA. The subgroup analysis was further conducted according to gender, age, and body mass index (BMI) Z-score.

## Methods

### Study population

This cross-sectional study used data from the NHANES 2005–2018. The NHANES is a nationally representative survey of nutrition and health condition in the United States, combining interviews and physical examinations, which are conducted by the National Center for Health Statistics (NCHS) (https://www.cdc.gov/nchs/nhanes/about_nhanes.htm). Written informed consent to participate in this survey was obtained from the participants or their legal guardians. The NHANES is approved by the NCHS Ethics Review Board (ERB) (https://www.cdc.gov/nchs/nhanes/irba98.htm). Ethical approval was exempt for this study because publicly available, de-identified data from the NHANES were used. Individuals aged 8–17 years in the NHANES 2005–2018 were included, whereas individuals without data on hypertension, fatty acid intake, BP, and covariates were excluded.

### Assessment of dietary intake of total fat and fatty acid

Dietary intake of total fat and fatty acid was evaluated via two 24-h dietary recall interviews and represented by the average intake from the two interviews in order to have a relatively complete estimate of usual dietary intake (15, 16). The first dietary recall interview was performed in person in the mobile examination center (MEC), and the second interview was performed via telephone 3–10 days later. In this study, fatty acids included total SFAs, total MUFAs, and total PUFAs (omega-3 and omega-6 PUFAs). Omega-3 PUFAs included octadecatrienoic acid (18:3), octadecatetraenoic acid (18:4), eicosapentaenoic acid (20:5), docosapentaenoic acid (22:5), and docosahexaenoic acid (22:6). Omega-6 PUFAs included octadecadienoic acid (18:2) and eicosatetraenoic acid (20:4). The omega-6/omega-3 ratio was calculated. The density of total fat and fatty acids was obtained by dividing the energy corresponding to the total fat and fatty acid intake (total fat and fatty acid intake × 9 calories/g × 1,000) by the total energy intake. This density was used to calculate the proportion of energy represented by total fat and fatty acids in total energy. Dietary intake of total fat and fatty acids showed the amount of total fat and fatty acids consumed.

### Assessment of hypertension

Participants were deemed as hypertensive if (1) they (aged ≥16 years) or their parent/guardian (aged <16 years) reported that they were diagnosed with hypertension irrespective of the BP value, (2) currently taking an antihypertensive medication irrespective of the BP value, or (3) classified as having elevated BP/hypertension according to the 2017 guideline from the American Academy of Pediatrics (AAP) (17, 18). For item (3), BP was measured in NHANES as follows: after the participant quietly rested in a seated position for 5 min and after the maximum inflation level (MIL) of the participant was determined; three consecutive BP readings were obtained. If a BP measurement was interrupted or incomplete, a fourth attempt was made. SBP and DBP were taken in the MEC, and their means were calculated. The BP examiners were certified for BP measurement via a training program from Shared Care Research and Education Consulting. When the examiners meet all the requirements of the training program, certification can be obtained (https://wwwn.cdc.gov/Nchs/Nhanes/2011-2012/BPX_G.htm), which provides quality assurance and quality control.

### Covariates

The following characteristics were also collected: age, sex, race (non-Hispanic white, non-Hispanic black, other), pediatric BMI Z-score, pediatric waist circumference Z-score, educational level (below the fifth grade, fifth–eighth grade, above the eighth grade), and intake of dietary calcium, potassium, sodium, protein, carbohydrate, and energy. Data on the intake of dietary calcium, potassium, sodium, protein, carbohydrate, and energy were obtained by averaging the intake from the two 24-h dietary recall interviews. The pediatric BMI Z-score was calculated using the BMI-for-age growth charts formulated by the Centers for Disease Control and Prevention (19). The pediatric waist circumference Z-score was calculated using the LMS tables for children and adolescents (20).

### Statistical analysis

Continuous variables were tested for normality using the Kolmogorov–Smirnov test. Continuous variables of normal distribution were reported as mean ± standard deviation (SD), and the *t*-test was used to compare the hypertensive and non-hypertensive groups. Continuous variables of skewed distribution were described by median and quartiles [M (Q_1_, Q_3_)], and intergroup comparisons were completed using the Wilcoxon rank-sum test. The *χ*^2^ test was utilized for intergroup comparison of categorical variables, which was described as the number of cases and the constituent ratio [*n* (%)]. Univariate logistic regression models were used to investigate the variables associated with hypertension, whereas multivariate logistic regression models were used to investigate the association of fatty acid intake/intensity with hypertension. In addition, the adjusted covariates were variables that were significantly associated with hypertension in the univariate regression. For the assessment of the association between fatty acid intake and hypertension, Model 1 was adjusted for age and sex, whereas Model 2 was adjusted for age, sex, race, BMI Z-score, waist circumference Z-score, educational level, and intake of dietary sodium, protein, and energy. In assessing the association between fatty acid density and hypertension, Model 1 was adjusted for age and sex, whereas Model 2 was adjusted for age, sex, race, BMI Z-score, waist circumference Z-score, educational level, and intake of dietary sodium. The subgroup analysis was performed according to age (8–12 years, 13–17 years) (21, 22), gender, and BMI Z-score (non-obese, obese) (19). Odds ratios (ORs) and 95% confidence intervals (CIs) were calculated. Complex sampling designs and weights were considered in all analyses. R 4.1.3 (Institute for Statistics and Mathematics, Vienna, Austria) was adopted for statistical analyses. Two-sided *P* < 0.05 denoted a statistically significant difference.

## Results

### Participant characteristics

**Figure 1:**
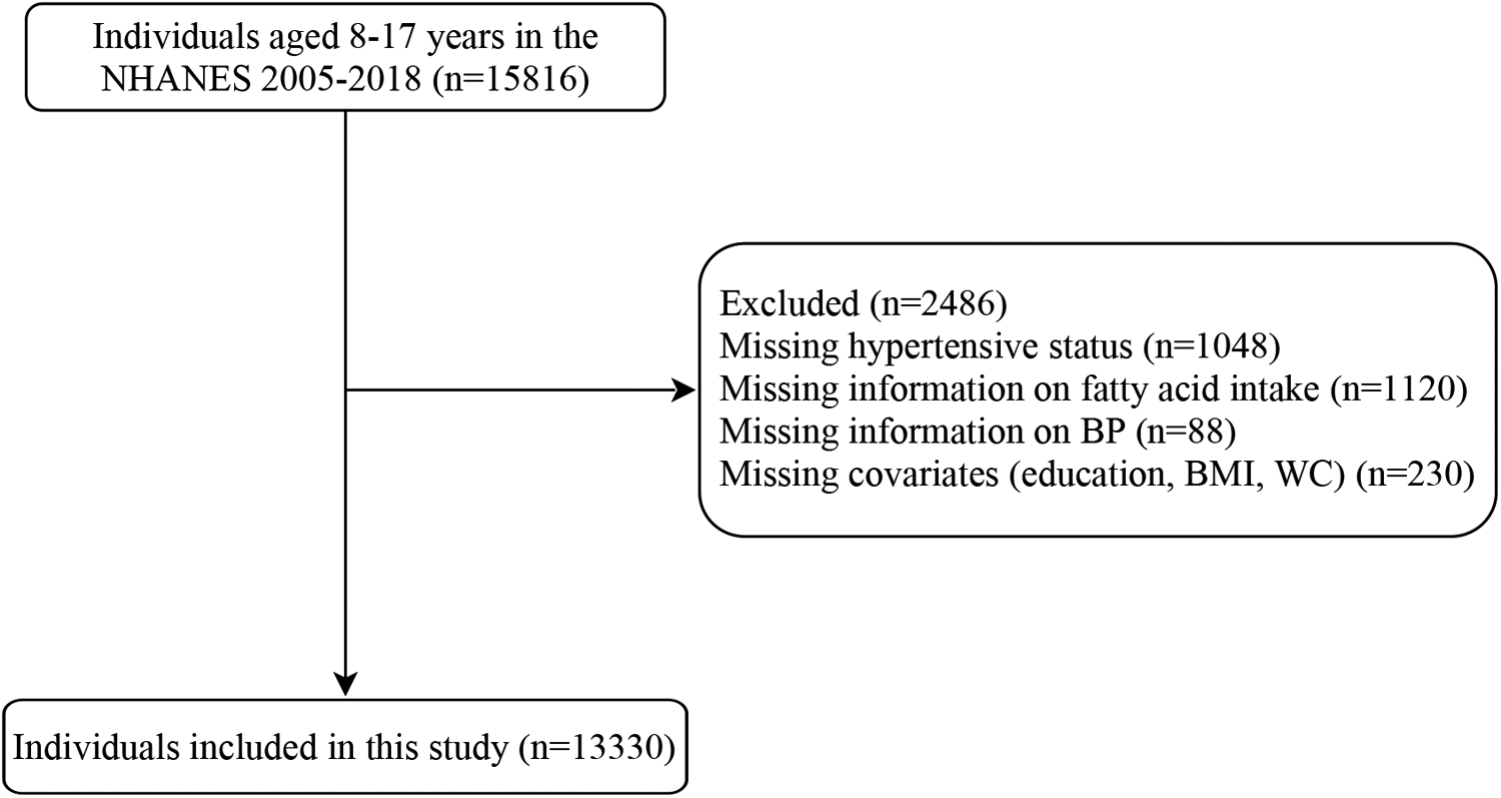
Flowchart of participant selection. WC, waist circumference.

After excluding individuals without data on hypertension (*n* = 1,048), fatty acid intake (*n* = 1,120), BP (*n* = 88), and covariates (educational level, BMI, and waist circumference) (*n* = 230), 13,330 subjects were included in this study, of which 11,614 were non- hypertensive (non-hypertensive group) and 1,716 were hypertensive (hypertensive group). Figure 1 presents the flowchart of how the participants are selected. The hypertensive group had significantly higher age, BMI Z-score; waist circumference Z-score; educational level; intake of dietary sodium, protein, carbohydrate, total fat, energy, total SFAs, and total MUFAs; and total MUFA density than the non-hypertensive group (all *P* < 0.05). Most of the hypertensive children and adolescents were male or non-Hispanic black compared with those who are non-hypertensive (both *P* < 0.05). The baseline characteristics of the included population are listed in Table 1.

**Table 1:** Baseline characteristics of the included population.

Variable	Non-hypertensive group (*n* = 11,614)	Hypertensive group (*n* = 1,716)	*P*
Age, year, mean ± SD	12.32 ± 2.84	13.27 ± 3.01	<0.001
Sex, *n* (%)			<0.001
Male	5,806 (49.99)	1,076 (62.70)	
Female	5,808 (50.01)	640 (37.30)	
Race, *n* (%)			<0.001
Non-Hispanic white	3,544 (30.51)	498 (29.02)	
Non-Hispanic black	2,882 (24.81)	545 (31.76)	
Other	5,188 (44.67)	673 (39.22)	
BMI Z-score, M (Q_1_, Q_3_)	0.71 (−0.09, 1.55)	1.50 (0.58, 2.16)	<0.001
Waist circumference Z-score, M (Q_1_, Q_3_)	0.50 (−0.19, 1.30)	1.17 (0.35, 1.89)	<0.001
Educational level, *n* (%)			<0.001
Below the fifth grade	4,823 (41.53)	540 (31.47)	
Fifth–eighth grade	4,012 (34.54)	481 (28.03)	
Above the eighth grade	2,779 (23.93)	695 (40.50)	
Calcium, gm, M (Q_1_, Q_3_)	913.25 (635.50, 1,246.50)	915.00 (607.75, 1,241.25)	0.602
Potassium, gm, M (Q_1_, Q_3_)	2,059.25 (1,575.00, 2,660.00)	2,033.50 (1,537.25, 2,700.75)	0.957
Sodium, gm, M (Q_1_, Q_3_)	2,995.75 (2,299.50, 3,844.00)	3,099.50 (2,394.00, 4,019.50)	<0.001
Energy, kcal, M (Q_1_, Q_3_)	1,877.25 (1,490.00, 2,362.00)	1,950.00 (1,514.25, 2,468.25)	<0.001
Protein, gm, M (Q_1_, Q_3_)	67.30 (51.63, 86.07)	68.91 (52.46, 91.78)	0.001
Carbohydrate, gm, M (Q_1_, Q_3_)	246.80 (193.14, 312.11)	253.92 (192.68, 332.58)	0.007
Total fat, gm, M (Q_1_, Q_3_)	69.73 (52.24, 91.51)	72.84 (53.06, 95.24)	<0.001
Total SFAs, gm, M (Q_1_, Q_3_)	23.76 (17.23, 31.74)	24.74 (17.66, 32.75)	0.006
Total MUFAs, gm, M (Q_1_, Q_3_)	24.23 (17.79, 32.23)	25.64 (18.45, 33.83)	<0.001
Total PUFAs, gm, M (Q_1_, Q_3_)	14.56 (10.38, 20.13)	14.88 (10.32, 21.18)	0.097
Protein, %, mean ± SD	14.68 ± 3.71	14.82 ± 3.78	0.164
Carbohydrate, %, mean ± SD	52.76 ± 7.70	52.44 ± 8.16	0.130
Total fat, %, mean ± SD	33.60 ± 6.31	33.65 ± 6.62	0.806
Total SFAs, %, mean ± SD	11.54 ± 2.93	11.48 ± 2.90	0.402
Total MUFAs, %, Mean ± SD	11.71 ± 2.68	11.86 ± 2.81	0.033
Total PUFAs, %, mean ± SD	6.97 (5.63, 8.68)	6.84 (5.46, 8.65)	0.110

### Association of fatty acid intake and density with hypertension

According to a univariate analysis, hypertension was slightly associated with older age, female sex, non-Hispanic black race, higher BMI Z-score, higher waist circumference Z-score, educational level of more than eighth grade, and higher intake of dietary sodium, protein, total fat, energy, total SFAs, and total MUFAs ( *P* < 0.05) (Table 2). After adjusting for age, sex, race, BMI Z-score, waist circumference Z-score, educational level, and intake of dietary sodium, protein, and energy, we found that a higher intake of total PUFA was associated with significantly lower odds of hypertension (OR = 0.85, 95% CI: 0.74–0.97, *P* = 0.018) (Table 3). No significant associations were found between the density of total SFAs, MUFAs, and PUFAs and the odds of hypertension after adjusting for age, sex, race, BMI Z-score, waist circumference Z-score, educational level, and intake of dietary sodium (all *P* > 0.05) (Table 4).

**Table 2:** Univariate analysis for variables associated with hypertension.

Variable	OR (95% CI)	*P*
Age	1.12 (1.07–1.16)	<0.001
Sex		
Male	Ref	
Female	0.59 (0.49–0.71)	<0.001
Race		
Non-Hispanic white	Ref	
Non-Hispanic black	1.42 (1.15–1.74)	0.001
Other	0.98 (0.80–1.21)	0.883
BMI Z-score	1.77 (1.61–1.95)	<0.001
Waist circumference Z-score	1.86 (1.68–2.05)	<0.001
Educational level		
Below the fifth grade	Ref	
Fifth–eighth grade	0.78 (0.60–1.01)	0.058
Above the eighth grade	1.90 (1.47–2.46)	<0.001
Calcium	0.99 (0.91–1.09)	0.879
Potassium	1.04 (0.94–1.14)	0.428
Sodium	1.15 (1.08–1.22)	<0.001
Energy	1.09 (1.01–1.19)	0.031
Protein (g)	1.11 (1.02–1.20)	0.013
Carbohydrate (g)	1.07 (0.97–1.17)	0.195
Total fat (g)	1.10 (1.02–1.18)	0.013
Total SFAs (g)	1.08 (1.01–1.15)	0.036
Total MUFAs (g)	1.13 (1.05–1.21)	0.001
Total PUFAs (g)	1.02 (0.94–1.11)	0.567
Protein (%)	1.04 (0.95–1.14)	0.404
Carbohydrate (%)	0.94 (0.85–1.03)	0.196
Total fat (%)	1.05 (0.94–1.16)	0.409
Total SFAs (%)	1.03 (0.93–1.14)	0.601
Total MUFAs (%)	1.10 (1.00–1.22)	0.050
Total PUFAs (%)	0.94 (0.85–1.04)	0.254

**Table 3:** Association between fatty acid intake and hypertension.

Variable	Model 1^a^		Model 2^b^	
	OR (95% CI)	*P*	OR (95% CI)	*P*
Total fat (g)	1.01 (0.94–1.08)	0.876	0.99 (0.78–1.26)	0.952
Total SFAs (g)	1.00 (0.93–1.07)	0.974	1.02 (0.85–1.23)	0.799
Total MUFAs (g)	1.04 (0.97–1.11)	0.268	1.15 (0.96–1.38)	0.124
Total PUFAs (g)	0.95 (0.88–1.03)	0.247	0.85 (0.74–0.97)	0.018

^a^ Model 1 adjusted for age and sex.

^b^ Model 2 adjusted for age, sex, race, BMI Z-score, waist circumference Z-score, educational level, and intake of dietary sodium, protein, and energy.

**Table 4:** Association between fatty acid density and hypertension.

Variable	Model 1^a^		Model 2^b^	
	OR (95% CI)	*P*	OR (95% CI)	*P*
Total fat (%)	1.04 (0.94–1.16)	0.440	1.00 (0.98–1.02)	0.929
Total SFAs (%)	1.03 (0.92–1.14)	0.633	1.01 (0.97–1.05)	0.634
Total MUFAs (%)	1.10 (1.00–1.21)	0.051	1.02 (0.99–1.06)	0.186
Total PUFAs (%)	0.95 (0.86–1.05)	0.298	0.96 (0.92–1.00)	0.074

^a^ Model 1 adjusted for age and sex.

^b^ Model 2 adjusted for age, sex, race, BMI Z-score, waist circumference Z-score, educational level, and intake of dietary sodium.

### Association of PUFA intake and density with hypertension

Increased intake of omega-3 (OR = 0.82, 95% CI: 0.72–0.93, *P* = 0.002) and omega-6 (OR = 0.86, 95% CI: 0.75–0.98, *P* = 0.025) PUFAs, octadecatrienoic acid (OR = 0.82, 95% CI: 0.72–0.93, *P* = 0.003), and octadecadienoic acid (OR = 0.86, 95% CI: 0.75–0.98, *P* = 0.025) was associated with significantly lower odds of hypertension, and individuals with higher omega-6/omega-3 ratio had significantly higher odds of hypertension (OR = 1.09, 95% CI: 1.02–1.17, *P* = 0.025), after adjusting for age, sex, race, BMI Z-score, waist circumference Z-score, educational level, and intake of dietary sodium, protein, and energy (Table 5). After controlling for age, sex, race, BMI Z-score, waist circumference Z-score, educational level, and intake of dietary sodium, we found that the density of omega-3 PUFAs (OR = 0.86, 95% CI: 0.78–0.95, *P* = 0.004) and octadecatrienoic acid (OR = 0.87, 95% CI: 0.78–0.96, *P* = 0.006) was inversely associated with the odds of hypertension, and omega-6/omega-3 ratio was positively associated with the odds of hypertension (OR = 1.09, 95% CI: 1.02–1.17, *P* = 0.012) (Table 6).

**Table 5:** Association between PUFA intake and hypertension.

Variable	Model 1^a^		Model 2^b^	
	OR (95% CI)	*P*	OR (95% CI)	*P*
Omega-3 PUFAs (g)	0.93 (0.86–1.00)	0.054	0.82 (0.72–0.93)	0.002
Omega-6 PUFAs (g)	0.96 (0.88–1.04)	0.280	0.86 (0.75–0.98)	0.025
Omega-6/omega-3	1.01 (0.99–1.03)	0.214	1.09 (1.02–1.17)	0.012
Octadecatrienoic acid (g)	0.93 (0.86–1.00)	0.059	0.82 (0.72–0.93)	0.003
Octadecatetraenoic acid (g)	1.00 (0.93–1.08)	0.969	1.00 (0.92–1.09)	0.920
Eicosapentaenoic acid (g)	0.99 (0.92–1.06)	0.673	0.99 (0.92–1.07)	0.792
Docosapentaenoic acid (g)	0.98 (0.90–1.05)	0.531	0.93 (0.85–1.03)	0.165
Docosahexaenoic acid (g)	0.97 (0.90–1.05)	0.498	0.97 (0.90–1.05)	0.494
Octadecadienoic acid (g)	0.96 (0.88–1.04)	0.273	0.86 (0.75–0.98)	0.025
Eicosatetraenoic acid (g)	1.04 (0.97–1.11)	0.251	1.02 (0.93–1.12)	0.692

^a^ Model 1 adjusted for age and sex.

^b^ Model 2 adjusted for age, sex, race, BMI Z-score, waist circumference Z-score, educational level, and intake of dietary sodium, protein, and energy.

**Table 6:** Association between PUFA density and hypertension.

Variable	Model 1^a^		Model 2^b^	
	OR (95% CI)	*P*	OR (95% CI)	*P*
Omega-3 PUFAs (%)	0.90 (0.83–0.99)	0.035	0.86 (0.78–0.95)	0.004
Omega-6 PUFAs (%)	0.95 (0.86–1.05)	0.342	0.91 (0.82–1.02)	0.095
Omega-6/omega-3	1.01 (0.99–1.03)	0.214	1.09 (1.02–1.17)	0.012
Octadecatrienoic acid (%)	0.69 (0.48–0.98)	0.042	0.87 (0.78–0.96)	0.006
Octadecatetraenoic acid (%)	1.02 (0.94–1.11)	0.631	1.02 (0.93–1.11)	0.719
Eicosapentaenoic acid (%)	0.97 (0.90–1.05)	0.450	0.97 (0.89–1.06)	0.476
Docosapentaenoic acid (%)	0.97 (0.88–1.07)	0.522	0.92 (0.83–1.02)	0.100
Docosahexaenoic acid (%)	0.95 (0.87–1.05)	0.310	0.94 (0.86–1.04)	0.216
Octadecadienoic acid (%)	0.95 (0.86–1.05)	0.335	0.91 (0.82–1.02)	0.095
Eicosatetraenoic acid (%)	1.05 (0.97–1.14)	0.250	0.99 (0.92–1.08)	0.871

^a^ Model 1 adjusted for age and sex.

^b^ Model 2 adjusted for age, sex, race, BMI Z-score, waist circumference Z-score, educational level, and intake of dietary sodium.

### Association of PUFA intake with hypertension by age, sex, and BMI Z-score

Increased intake of PUFAs (OR = 0.67, 95% CI: 0.49–0.91, *P* = 0.012), omega-3 (OR = 0.70, 95% CI: 0.53–0.92, *P* = 0.010) and omega-6 (OR = 0.68, 95% CI: 0.50–0.92, *P* = 0.013) PUFAs, octadecatrienoic acid (OR = 0.67, 95% CI: 0.50–0.90, *P* = 0.008), and octadecadienoic acid (OR = 0.68, 95% CI: 0.50–0.92, *P* = 0.012) was correlated with significantly lower odds of hypertension in participants aged 8–12 years. Increased intake of omega-3 PUFAs (OR = 0.70, 95% CI: 0.54–0.90, *P* = 0.006) and octadecatrienoic acid (OR = 0.69, 95% CI: 0.53–0.89, *P* = 0.005) was related to significantly lower odds of hypertension among females. Non-obese children who had a higher intake of omega-3 PUFAs (OR = 0.82, 95% CI: 0.71–0.95, *P* = 0.009) and octadecatrienoic acid (OR = 0.83, 95% CI: 0.72–0.95, *P* = 0.010) exhibited significantly lower odds of hypertension, and those with higher omega-6/omega-3 ratio had significantly higher odds of hypertension (OR = 1.22, 95% CI: 1.05–1.42, *P* = 0.011). For obese children, increased intake of PUFAs (OR = 0.76, 95% CI: 0.61–0.96, *P* = 0.022), omega-6 PUFAs (OR = 0.77, 95% CI: 0.61–0.96, *P* = 0.019), and octadecadienoic acid (OR = 0.77, 95% CI: 0.61–0.96, *P* = 0.019) was associated with lower odds of hypertension (Table 7).

**Table 7:** Association between PUFA intake and hypertension by age, sex, and BMI Z-score.

Variable	OR (95% CI)	*P*	OR (95% CI)	*P*
Age, years	8–12		13–17	
PUFAs (g)	0.67 (0.49–0.91)	0.012	0.93 (0.80–1.08)	0.354
Omega-3 PUFAs (g)	0.70 (0.53–0.92)	0.010	0.88 (0.77–1.01)	0.065
Omega-6 PUFAs (g)	0.68 (0.50–0.92)	0.013	0.94 (0.81–1.10)	0.426
Omega-6/omega-3	1.07 (0.91–1.26)	0.379	1.09 (0.99–1.19)	0.067
Octadecatrienoic acid (g)	0.67 (0.50–0.90)	0.008	0.89 (0.78–1.02)	0.099
Octadecatetraenoic acid (g)	1.06 (0.93–1.21)	0.382	0.99 (0.88–1.10)	0.800
Eicosapentaenoic acid (g)	1.09 (0.96–1.25)	0.184	0.94 (0.85–1.05)	0.279
Docosapentaenoic acid (g)	1.00 (0.83–1.21)	0.994	0.90 (0.80–1.02)	0.087
Docosahexaenoic acid (g)	1.08 (0.95–1.24)	0.233	0.93 (0.83–1.04)	0.202
Octadecadienoic acid (g)	0.68 (0.50–0.92)	0.012	0.94 (0.81–1.09)	0.421
Eicosatetraenoic acid (g)	0.97 (0.79–1.20)	0.801	1.04 (0.93–1.18)	0.468
Sex	Male		Female	
PUFAs (g)	0.88 (0.75–1.03)	0.110	0.82 (0.63–1.06)	0.130
Omega-3 PUFAs (g)	0.90 (0.77–1.06)	0.197	0.70 (0.54–0.90)	0.006
Omega-6 PUFAs (g)	0.88 (0.75–1.03)	0.102	0.85 (0.65–1.10)	0.214
Omega-6/omega-3	1.03 (0.93–1.14)	0.551	1.19 (0.99–1.43)	0.065
Octadecatrienoic acid (g)	0.91 (0.77–1.07)	0.236	0.69 (0.53–0.89)	0.005
Octadecatetraenoic acid (g)	1.00 (0.91–1.10)	0.968	1.01 (0.86–1.19)	0.863
Eicosapentaenoic acid (g)	0.98 (0.89–1.07)	0.631	1.04 (0.90–1.20)	0.591
Docosapentaenoic acid (g)	0.92 (0.82–1.04)	0.192	0.95 (0.78–1.16)	0.606
Docosahexaenoic acid (g)	0.97 (0.88–1.06)	0.483	1.01 (0.86–1.18)	0.929
Octadecadienoic acid (g)	0.88 (0.75–1.03)	0.099	0.85 (0.65–1.10)	0.215
Eicosatetraenoic acid (g)	1.05 (0.93–1.18)	0.450	0.97 (0.79–1.20)	0.778
BMI Z-score	Non-obese		Obese	** **
PUFAs (g)	0.93 (0.78–1.11)	0.424	0.76 (0.61–0.96)	0.022
Omega-3 PUFAs (g)	0.82 (0.71–0.95)	0.009	0.83 (0.67–1.03)	0.083
Omega-6 PUFAs (g)	0.95 (0.80–1.14)	0.573	0.77 (0.61–0.96)	0.019
Omega-6/omega-3	1.22 (1.05–1.42)	0.011	1.00 (0.89–1.13)	0.976
Octadecatrienoic acid (g)	0.83 (0.72–0.95)	0.010	0.83 (0.66–1.03)	0.089
Octadecatetraenoic acid (g)	0.98 (0.88–1.09)	0.724	1.04 (0.91–1.19)	0.551
Eicosapentaenoic acid (g)	0.96 (0.86–1.08)	0.482	1.01 (0.92–1.11)	0.781
Docosapentaenoic acid (g)	1.00 (0.89–1.12)	0.978	0.87 (0.73–1.02)	0.093
Docosahexaenoic acid (g)	0.97 (0.86–1.09)	0.629	0.98 (0.89–1.08)	0.668
Octadecadienoic acid (g)	0.95 (0.79–1.13)	0.557	0.77 (0.61–0.96)	0.019
Eicosatetraenoic acid (g)	1.12 (0.99–1.27)	0.067	0.89 (0.74–1.08)	0.237

For age groups, the multivariate model adjusted for sex, race, BMI Z-score, waist circumference Z-score, educational level, and intake of dietary sodium, protein, and energy. For sex groups, the multivariate model adjusted for age, race, BMI Z-score, waist circumference Z-score, educational level, and intake of dietary sodium, protein, and energy. For BMI Z-score groups, the multivariate model adjusted for age, sex, race, waist circumference Z-score, educational level, and intake of dietary sodium, protein, and energy.

### Association of PUFA density with hypertension by age, sex, and BMI Z-score

Among participants aged 8–12 years, higher density of PUFAs (OR = 0.91, 95% CI: 0.84–0.99, *P* = 0.027), omega-3 (OR = 0.80, 95% CI: 0.65–0.98, *P* = 0.030) and omega-6 (OR = 0.80, 95% CI: 0.65–0.98, *P* = 0.029) PUFAs, octadecatrienoic acid (OR = 0.78, 95% CI: 0.62–0.97, *P* = 0.025), and octadecadienoic acid (OR = 0.80, 95% CI: 0.66–0.98, *P* = 0.029) was associated with significantly lower odds of hypertension. Increased density of docosapentaenoic acid was associated with significantly lower odds of hypertension in males (OR = 0.88, 95% CI: 0.78–0.99, *P* = 0.031). Higher density of omega-3 PUFAs (OR = 0.82, 95% CI: 0.70–0.96, *P* = 0.014) and octadecatrienoic acid (OR = 0.81, 95% CI: 0.69–0.95, *P* = 0.011) was associated with significantly lower odds of hypertension, and omega-6/omega-3 ratio was positively correlated with the odds of hypertension (OR = 1.19, 95% CI: 1.00–1.42, *P* = 0.048) in females. Regarding non-obese children, higher density of omega-3 PUFAs (OR = 0.87, 95% CI: 0.79–0.96, *P* = 0.006) and octadecatrienoic acid (OR = 0.88, 95% CI: 0.80–0.96, *P* = 0.007) was associated with significantly lower odds of hypertension, and increased omega-6/omega-3 ratio was associated with significantly higher odds of hypertension (OR = 1.22, 95% CI: 1.05–1.42, *P* = 0.009). No significant association was observed between PUFA density and hypertension in obese children (Table 8).

**Table 8:** Association between PUFA density and hypertension by age, sex, and BMI Z-score.

	OR (95% CI)	*P*	OR (95% CI)	*P*
Age, years	8–12		13–17	
PUFAs (%)	0.91 (0.84–0.99)	0.027	0.99 (0.94–1.03)	0.589
Omega-3 PUFAs (%)	0.80 (0.65–0.98)	0.030	0.90 (0.80–1.01)	0.082
Omega-6 PUFAs (%)	0.80 (0.65–0.98)	0.029	0.97 (0.86–1.10)	0.671
Omega-6/omega-3	1.09 (0.93–1.29)	0.271	1.08 (0.99–1.18)	0.079
Octadecatrienoic acid (%)	0.78 (0.62–0.97)	0.025	0.92 (0.82–1.03)	0.130
Octadecatetraenoic acid (%)	1.07 (0.92–1.26)	0.379	1.00 (0.90–1.11)	0.976
Eicosapentaenoic acid (%)	1.07 (0.95–1.21)	0.261	0.89 (0.77–1.02)	0.094
Docosapentaenoic acid (%)	0.96 (0.81–1.13)	0.636	0.89 (0.79–1.00)	0.057
Docosahexaenoic acid (%)	1.04 (0.92–1.18)	0.511	0.88 (0.76–1.00)	0.056
Octadecadienoic acid (%)	0.80 (0.66–0.98)	0.029	0.97 (0.86–1.10)	0.665
Eicosatetraenoic acid (%)	0.92 (0.80–1.07)	0.267	1.03 (0.93–1.14)	0.597
Sex	Male		Female	
PUFAs (%)	0.97 (0.92–1.03)	0.314	0.96 (0.90–1.02)	0.193
Omega-3 PUFAs (%)	0.92 (0.79–1.06)	0.251	0.82 (0.70–0.96)	0.014
Omega-6 PUFAs (%)	0.93 (0.82–1.07)	0.305	0.91 (0.77–1.07)	0.270
Omega-6/omega-3	1.03 (0.94–1.14)	0.503	1.19 (1.00–1.42)	0.048
Octadecatrienoic acid (%)	0.94 (0.81–1.08)	0.375	0.81 (0.69–0.95)	0.011
Octadecatetraenoic acid (%)	1.00 (0.90–1.10)	0.927	1.04 (0.92–1.19)	0.512
Eicosapentaenoic acid (%)	0.91 (0.81–1.03)	0.122	1.05 (0.93–1.19)	0.440
Docosapentaenoic acid (%)	0.88 (0.78–0.99)	0.031	0.97 (0.82–1.14)	0.676
Docosahexaenoic acid (%)	0.90 (0.80–1.02)	0.092	1.00 (0.87–1.15)	0.963
Octadecadienoic acid (%)	0.93 (0.82–1.06)	0.300	0.91 (0.77–1.08)	0.274
Eicosatetraenoic acid (%)	1.02 (0.91–1.14)	0.726	0.95 (0.83–1.08)	0.438
BMI Z-score	Non-obese		Obese	** **
PUFAs (g)	0.98 (0.94–1.03)	0.470	0.94 (0.87–1.01)	0.102
Omega-3 PUFAs (g)	0.87 (0.79–0.96)	0.006	0.86 (0.70–1.06)	0.149
Omega-6 PUFAs (g)	0.97 (0.86–1.09)	0.606	0.85 (0.70–1.03)	0.098
Omega-6/omega-3	1.22 (1.05–1.42)	0.009	1.00 (0.89–1.13)	0.987
Octadecatrienoic acid (g)	0.88 (0.80–0.96)	0.007	0.86 (0.70–1.06)	0.159
Octadecatetraenoic acid (g)	1.03 (0.92–1.14)	0.629	1.01 (0.88–1.16)	0.890
Eicosapentaenoic acid (g)	0.92 (0.81–1.04)	0.158	1.01 (0.90–1.13)	0.851
Docosapentaenoic acid (g)	0.95 (0.85–1.06)	0.369	0.89 (0.73–1.08)	0.220
Docosahexaenoic acid (g)	0.93 (0.82–1.05)	0.237	0.96 (0.85–1.09)	0.518
Octadecadienoic acid (g)	0.97 (0.86–1.09)	0.598	0.85 (0.70–1.03)	0.099
Eicosatetraenoic acid (g)	1.04 (0.95–1.13)	0.428	0.94 (0.79–1.12)	0.498

For age groups, the multivariate model adjusted for sex, race, BMI Z-score, waist circumference Z-score, educational level, and intake of dietary sodium. For sex groups, the multivariate model adjusted for age, race, BMI Z-score, waist circumference Z-score, educational level, and intake of dietary sodium. For BMI Z-score groups, the multivariate model adjusted for age, sex, race, waist circumference Z-score, educational level, and intake of dietary sodium, protein, and energy.

## Discussion

This study first investigated the association between dietary fatty acid intake and density and hypertension in children and adolescents. In this study, fatty acids included total SFAs, total MUFAs, and total PUFAs (omega-3 and omega-6 PUFAs), and the relationship between omega-6/omega-3 ratio and hypertension was evaluated. We found that total PUFA intake was inversely associated with the odds of hypertension. Increased intake of omega-3 and omega-6 PUFAs, octadecatrienoic acid, and octadecadienoic acid was associated with significantly lower odds of hypertension, and higher density of omega-3 PUFAs and octadecatrienoic acid was associated with significantly lower odds of hypertension. Individuals with higher omega-6/omega-3 ratio had significantly higher odds of hypertension.

According to a review by Couch and Daniels, several studies focused on the relationship between dietary fatty acids and BP in children (23). In a previous cross-sectional study, the association between PUFA intake and BP (SBP and DBP) was assessed among adolescents (11). Tang et al. (12) illustrated that palmitoleic acid was negatively correlated with the risk of primary hypertension in children and adolescents. The literature on the association between dietary fatty acid and hypertension in children and adolescents was limited. In this study, total PUFA intake was found to be inversely associated with the odds of hypertension among children and adolescents. Ni et al. (24) proposed that the intake of total PUFAs, omega-3 fatty acids, and omega-6 fatty acids was associated with a lower prevalence of hypertension in American adults. A regular dietary intake of omega-6 fatty acid may contribute to the prevention and treatment of hypertension in a healthy general population, based on a research in Japanese people aged ≥40 years (25). Further, we demonstrated that children and adolescents with higher intake of omega-3 and omega-6 PUFAs had lower odds of hypertension. In particular, intake of octadecatrienoic acid and octadecadienoic acid was negatively associated with the odds of hypertension among children and adolescents. Based on existing studies, a randomized controlled trial reported that dietary supplementation with long-chain PUFAs during infancy was associated with lower BP in later childhood (26). Simons-Morton et al. (27) demonstrated the important role of total fat in the BP level of 8- to 11-year-old children with increased levels of low-density lipoprotein cholesterol. Dietary intake of *α*-linolenic acid, a plant-derived omega-3 PUFA, was found to be epidemiologically associated with a lower prevalence of hypertension (28). Djoussé et al. illustrated the association between the dietary intake of linolenic acid and a reduced prevalence of hypertension and decreased SBP (29). Higher plasma levels of linoleic acid and *α*-linolenic acid were shown to be significantly related to a lower prevalence of hypertension in Japanese men (30). BP was decreased after linoleic acid treatment (31). This study also identified an inverse association of the density of dietary omega-3 PUFAs and octadecatrienoic acid with the odds of hypertension for the first time and revealed that children and adolescents with higher omega-6/omega-3 ratio in diets had significantly higher odds of hypertension. It has been suggested that a lower omega-6/omega-3 ratio of fatty acids is associated with a reduced risk of chronic diseases (32). The positive results for lower density of dietary omega-3 PUFAs and octadecatrienoic acid indicated that children and adolescents who consume more omega-3 PUFAs and octadecatrienoic acid may have lower odds of hypertension when their total energy intake is the same. However, this result requires more evidence to confirm. We also illustrated that the associations of dietary PUFAs, omega-3 and omega-6 fatty acids, and omega-6/omega-3 ratio with hypertension varied across children and adolescents of different ages, genders, and BMI Z-scores. Age and gender were reported to be correlated with hypertension, and high intake of long-chain omega-3 PUFAs was related to reduced obesity-relevant cardiovascular risk factors (33). Obesity was the main cause of hypertension (34). Machate et al. (35) showed that diets rich in omega-3 PUFAs were associated with reduction or prevention of fat accumulation in the adipose tissue, obesity, and hypertension. Future studies are needed to verify our findings and investigate the underlying mechanisms among the pediatric population. Guardians may pay attention to specific PUFA intake of children aged 8–12 years (male, female, obese, and non-obese children) to facilitate the management of hypertension under the guidance and advice provided by clinicians.

PUFAs have the following physiological functions: (1) esterification of cholesterol and lowering blood cholesterol and triglyceride; (2) reducing platelet aggregation and thrombosis; and (3) reducing blood viscosity, improving blood microcirculation, and the like (10). Communities, schools, and guardians could actively popularize the benefits of PUFA to children and adolescents to increase their awareness of reasonable intake of PUFA, with dietary advice given by dietitians. Children and adolescents may increase their PUFA intake by consuming more walnuts, fish (e.g., salmon, tuna), oils (e.g., soybean oil, canola oil), dietary supplements, etc., to obtain the benefits (36). Hypertension is often featured by impaired vasodilation, involving dysfunction of multiple vasodilatory mechanisms. Intake of omega-3 PUFAs can reduce BP and vasodilation (37). The vasodilatory effects of omega-3 PUFAs on vascular smooth muscle cells (VSMCs) are mediated through the opening of large-conductance calcium-activated potassium (BKCa) channels, ATP-sensitive potassium (KATP) channels, and possible members of the voltage-activated potassium channel K_v_7 family, leading to hyperpolarization and relaxation (37). It is a well-known fact that inflammatory effects play an important role in the occurrence and development of hypertension. This anti-inflammatory effect can also explain the mechanism between omega-3 fatty acid and the lower odds of hypertension (38, 39). In addition, omega-3 and omega-6 fatty acids can act as signal molecules on peroxisome proliferator-activated receptors (PPARs), which can modulate metabolic processes, including lipid and glucose metabolism, inflammation, or oxidative stress (40). Antioxidant effects, suppression of thromboxane production, and reduction of plasma homocysteine levels may also explain the relation of omega-3 and omega-6 fatty acid intake to the odds of hypertension (41, 42). The positive association between omega-6/omega-3 ratio and odds of hypertension may be that omega-6 PUFA arachidonic acid contributes to fatty acids present in the membrane phospholipids of cells involved in inflammation. Arachidonic acid is a precursor to many potent pro-inflammatory mediators and vasoconstrictor prostaglandins (43, 44). Some researchers recommend lowering the omega-6/omega-3 ratio to reduce adverse effects caused by excess arachidonic acid and its eicosanoid products, such as inflammation (45).

The present study used a nationally representative sample (*n* = 13,330) from the NHANES to assess the association between dietary fatty acids and hypertension among children and adolescents. Based on the findings, the PUFA intake level of children and adolescents could be closely monitored, and thus timely interventions may be provided to reduce the odds of hypertension. Some limitations are noted. First, due to the cross-sectional design of this study, causality could not be determined, and reverse causality may be possible. Second, confounding factors cannot be fully controlled, and the database only records five kinds of omega-3 fatty acids and two kinds of omega-6 fatty acids, which may influence the accuracy of the results. Third, a possible recall bias in the 24-h dietary recall interviews may result in the associations attenuated toward the null. The obtained fatty acid intake may not represent the habitual intake of children and adolescents, and this problem was alleviated by averaging two intakes from the two interviews. In addition, data collected from the interviews were used to define hypertension and may influence the accuracy of the findings. Finally, this study was conducted based on the data from the US population, and its generalizability may be limited in other populations.

## Conclusion

Total PUFA intake was negatively associated with the odds of hypertension in children and adolescents. Both increased intake of omega-3 and omega-6 PUFAs, octadecatrienoic acid, and octadecadienoic acid and higher density of omega-3 PUFAs and octadecatrienoic acid were associated with lower odds of hypertension. Individuals with higher omega-6/omega-3 ratio had significantly higher odds of hypertension. Future well-designed studies are warranted to support these findings.

## Data Availability

Publicly available datasets were analyzed in this study. These data can be found in the NHANES database: https://www.cdc.gov/nchs/nhanes/index.html.
